# The SKP2‐p27 axis defines susceptibility to cell death upon CHK1 inhibition

**DOI:** 10.1002/1878-0261.13264

**Published:** 2022-07-07

**Authors:** Michael Lohmüller, Bernhard F. Roeck, Tamas G. Szabo, Marina A. Schapfl, Fragka Pegka, Sebastian Herzog, Andreas Villunger, Fabian Schuler

**Affiliations:** ^1^ Institute for Developmental Immunology, Biocenter Medical University of Innsbruck Austria; ^2^ Institute for Medical Biochemistry, Biocenter Medical University of Innsbruck Austria

**Keywords:** apoptosis, cell cycle, CHK1 inhibition, DNA‐damage, p27, SKP2

## Abstract

Checkpoint kinase 1 (CHK1; encoded by *CHEK1*) is an essential gene that monitors DNA replication fidelity and prevents mitotic entry in the presence of under‐replicated DNA or exogenous DNA damage. Cancer cells deficient in p53 tumor suppressor function reportedly develop a strong dependency on CHK1 for proper cell cycle progression and maintenance of genome integrity, sparking interest in developing kinase inhibitors. Pharmacological inhibition of CHK1 triggers B‐Cell CLL/Lymphoma 2 (BCL2)‐regulated cell death in malignant cells largely independently of p53, and has been suggested to kill p53‐deficient cancer cells even more effectively. Next to p53 status, our knowledge about factors predicting cancer cell responsiveness to CHK1 inhibitors is limited. Here, we conducted a genome‐wide CRISPR/Cas9‐based loss‐of‐function screen to identify genes defining sensitivity to chemical CHK1 inhibitors. Next to the proapoptotic BCL2 family member, BCL2 Binding Component 3 (BBC3; also known as PUMA), the F‐box protein S‐phase Kinase‐Associated Protein 2 (SKP2) was validated to tune the cellular response to CHK1 inhibition. SKP2 is best known for degradation of the Cyclin‐dependent Kinase Inhibitor 1B (CDKN1B; also known as p27), thereby promoting G1‐S transition and cell cycle progression in response to mitogens. Loss of SKP2 resulted in the predicted increase in p27 protein levels, coinciding with reduced DNA damage upon CHK1‐inhibitor treatment and reduced cell death in S‐phase. Conversely, overexpression of SKP2, which consequently results in reduced p27 protein levels, enhanced cell death susceptibility to CHK1 inhibition. We propose that assessing SKP2 and p27 expression levels in human malignancies will help to predict the responsiveness to CHK1‐inhibitor treatment.

## Graphical abstract

Checkpoint kinase 1 (CHK1) inhibition promotes cell death by the induction of DNA‐damage, leading to BCL2‐regulated apoptosis involving the BH3‐only protein PUMA/BBC3. Susceptibility of cancer cells to CHK1 inhibitors is fine‐tuned by cellular levels of the cyclin dependent kinase inhibitor, p27, and its degrader, SKP2. Assessment of p27/SKP2 expression levels may help to stratify patients for CHK1 inhibitor‐based therapies.

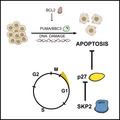

**Abbreviations**
BCL2: B‐cell CLL/lymphoma 2
CHK1: checkpoint kinase 1
CDKN1B: cyclin‐dependent kinase inhibitor 1B
SKP2: S‐phase kinase‐associated protein 2
BBC3: BCL2 binding component 3
PUMA: P53‐upregulated mediator of apoptosis
CDK: cyclin‐dependent kinase

## 1 Introduction

Checkpoint kinase 1 *(CHEK1/CHK1)* is a key component of the DNA damage response (DDR) machinery, able to react to single‐strand (ss) DNA breaks generated in response to genotoxic stress, replication errors, or replication fork stalling [1, 2]. ssDNA lesions are decorated by the Replication Protein A (RPA) and recognized by Ataxia telangiectasia and Rad3‐related (ATR) kinase that phosphorylates CHK1 on Ser^317^ and Ser^345^ for activation [2, 3]. In contrast, autophosphorylation on Ser^296^ controls its levels in steady state by regulating its proteasomal degradation [4]. CHK1 was shown to restrict cyclin‐dependent kinase (CDK) activity to slow down S‐phase progression and to prevent premature entry from G2 into mitosis in the presence of DNA damage. To do so, CHK1 targets CDC25A for degradation to inhibit cyclin‐dependent kinase 2 (CDK2)/cyclin E/A complexes, leading to reduced DNA synthesis [5, 6]. DNA damage causes CHK1 also to activate WEE1 kinase and block CDC25C phosphatase, thereby preventing mitotic entry by enhancing Tyr^15^ phosphorylation on CDK1/CDC2 causing its cytoplasmic retention [7, 8, 9]. In addition, activation of the tumor suppressor protein p53 by CHK1‐dependent phosphorylation enforces the restriction of cell cycle progression upon DNA damage [10, 11]. Accordingly, cancer cells with dysfunctional p53 were shown to develop a dependency on CHK1 for cell cycle control and survival [12]. Hence inhibiting CHK1 has turned out a promising strategy to kill cancer cells with mutant or deleted p53 [13, 14, 15].

Of note, several solid tumor entities show high CHK1 expression, potentially because oncogene‐driven replication stress and associated DNA damage are unphysiologically high in cancer [16, 17]. In line with these findings, highest mRNA levels of *CHK1* are found in fast proliferating lymphoma and leukemia, such as Burkitt lymphoma and pre‐B acute lymphoblastic leukemia, suggesting that high CHK1 activity is needed to balance replication stress caused by deregulated MYC or other oncogenic events that drive extensive proliferation [18]. Consistently, *Eμ‐MYC*‐driven B‐cell lymphomagenesis in mice depends on CHK1 [19] and tumors from these mice are highly responsive to CHK1 inhibitors [20], as are several human blood cancer cell lines, including those from Burkitt and Diffuse Large B‐cell Lymphomas (DLBCL) [19, 21]. These respond well to CHK1 inhibition alone, or when combined with antimetabolites or ATR inhibitors [13, 20, 21]. Similarly, AML patients with high CHK1 expression show poor prognosis and high relapse rates potentially due to increased S‐phase proficiency counteracting cytarabine treatment. Co‐treatment with the phase II clinical trial CHK1‐inhibitor, SCH900776, dramatically increased the effect of cytarabine treatment on AML [18]. Together these studies show that CHK1i can be considered a valid strategy to treat cancers with either MYC amplifications or defective p53. Consistent with this idea, a recent study conducting an unbiased small molecule drug screen in medulloblastoma cell lines that frequently harbor these genetic alterations uncovered CHK1 inhibitors to be highly synergistic in combination with standard of care therapy [22].

To widen the therapeutic potential of CHK1‐inhibitors it is important to understand cell death and resistance mechanisms that define the overall response and efficacy. Little information exists on how CHK1i treatment initiates tumor cell death and which genetic factors define inhibitor potency. Inhibitor binding causes CHK1 accumulation, due to impaired autophosphorylation on Ser^296^ that usually targets CHK1 to proteasomal degradation [4]. A recent study identified FAM122 phosphatase as a critical regulator of cancer cell susceptibility to CHK1i, as FAM122 is inhibited by CHK1 in order to stabilize WEE1 kinase activity [23]. We have shown that CHK1i promotes DNA damage that elicits mitochondrial apoptosis and caspase activation, which is controlled by pro‐ and anti‐apoptotic BCL2 family members [19, 24]. Overexpression of anti‐apoptotic BCL2 or MCL1 prevents CHK1i‐induced caspase activation and cell death in normal and transformed leukocytes nearly as potently as the combined loss of the apoptotic effector proteins BAX and BAK. Activation of BAX and BAK upon CHK1 inhibition involves a sub‐set of BH3‐only proteins, namely, BIM, PUMA/BBC3, and NOXA/PMAIP. Of note, p53 appears largely dispensable for cell death initiation in immortalized hematopoietic progenitor cells upon CHK1i treatment, despite being stabilized [19, 24]. This finding is in line with earlier studies reporting that loss of p53 fails to rescue the embryonic lethality caused by CHK1 deficiency or inhibitor‐induced cell death [3, 25]. The latter seems somewhat surprising, given that PUMA and NOXA are established p53 targets and needed to execute p53‐induced apoptosis in response to DNA damage [26, 27]. Yet, these findings also show that additional regulators and effectors of CHK1i‐induced apoptosis remain to be identified.

In order to gain additional insights into CHK1i‐induced cell death, we conducted a genome‐wide CRISPR/Cas9 loss‐of‐function screen where S‐Phase Kinase Associated Protein 2 (SKP2), best known for its ability to control the degradation of the CDK‐inhibitor, p27 (CDKN1B), emerged as the top hit [28, 29]. Loss of the E3‐ligase SKP2 renders mouse and human hematopoietic cancer cells less susceptible to CHK1‐inhibition. Of note, the protective effect relies on p27 stabilization and subsequent cell death inhibition. High levels of p27 trigger a reduction of CHK1i‐induced DNA damage, leading to an overall reduction of BCL2‐regulated apoptosis. Consistently, reduced levels of p27 as a result of SKP2 overexpression sensitizes cells to CHK1 inhibition. Our findings indicate that the SKP2‐p27 axis defines susceptibility to CHK1i‐induced apoptosis. Hence, we propose to assess SKP2 and p27 expression levels to stratify cancer patients considered for CHK1‐inhibitor treatment.

## 2 Materials and methods

### 2.1 Cell lines, tissue culture, and reagents

All cell lines used were cultured in a humidified atmosphere containing 7.5% CO_2_ as previously described [24]. Nalm6 cells (RRID: CVCL_0092; DSMZ, ACC128) were cultured in RPMI‐1640 medium (Sigma‐Aldrich Handels GmbH, Vienna, Austria, R0883) supplemented with 10% FCS (Thermo Fisher Scientific, Vienna, Austria, 10 270–106), 2 mm l‐glutamine (Sigma, G7513), 100 U·mL^−1^ penicillin, 100 μg·mL^−1^ streptomycin (Sigma, P0781). Murine Baf3 pro B cells (RRID: CVCL_0161, DMSZ, ACC300) and CRISPR knockout derivatives generated in our lab were cultured in RPMI‐1640 medium supplemented with 7.5% FCS (Thermo Fisher Scientific, 10 270–106), 2 mm l‐glutamine (Sigma, G7513), 100 U·mL^−1^ penicillin, 100 μg·mL^−1^ streptomycin (Sigma, P0781) and 2% of supernatant obtained from WEHI‐231 cells (RRID: CVCL_0577) secreting IL3. Hoxb8^FLT3^ bone marrow progenitor cells [24] were cultured in RPMI‐1640 (Sigma‐Aldrich, R0883) supplemented with 10% FCS (Thermo Fisher Scientific, 10 270–106), 2 mm l‐glutamine (Sigma, G7513), 100 U·mL^−1^ penicillin, 100 μg·mL^−1^ streptomycin (Sigma, P0781), 1 μm beta‐estradiol and 5% supernatant of FLT3 ligand‐expressing B16 melanoma cells. Human embryonic kidney (HEK) 293T cells (RRID: CVCL_0063; DMSZ, ACC635) were cultured in DMEM (Sigma‐Aldrich, D5671) supplemented with 10% FCS (Thermo Fisher Scientific, 10 270–106) 2 mm l‐glutamine (Sigma, G7513), 100 U·mL^−1^ penicillin and 100 μg·mL^−1^ streptomycin (Sigma, P0781).

CHK1 inhibitors PF‐477736 (Selleckchem, S2fh3904) or CHIR‐124 (Selleck Chemicals GmbH, Planegg, Germany, S2683) were dissolved in DMSO as recommended by the manufacturer; 10 mm stocks were used and stored at −80 °C. Aliquots were stored at −20 °C (Table 1).

**Table 1:** sgRNA sequences

sgRNA name	Sequence 5′–3′	Target gene
Bax_1_Fwd	caccgCAACTTCAACTGGGGCCGCG	*Bax* (mouse)
Bax_1_Rev	aaacCGCGGCCCCAGTTGAAGTTGc	
Bax_2_Fwd	caccgAGCGAGTGTCTCCGGCGAAT	
Bax_2_Rev	aaacATTCGCCGGAGACACTCGCTc	
Bak_1_Fwd	caccGGAACTCTGTGTCGTAGCGC	*Bak* (mouse)
Bak_1_Rev	aaacGCGCTACGACACAGAGTTCC	
Bbc3_1_Fwd	caccGCCGCTCGTACTGCGCGTTG	*Bbc3* (mouse)
Bbc3_1_Rev	aaacCAACGCGCAGTACGAGCGGC	
Bbc3_2_Fwd	caccGGGCACTCACCGTCCGGGCG	
Bbc3_2_Rev	aaacCGCCCGGACGGTGAGTGCCC	
Skp2_1_Fwd	caccgTTGCCCTTGACTCGTTTCCT	*Skp2* (mouse)
Skp2_1_Rev	aaacAGGAAACGAGTCAAGGGCAAc	
Skp2_2_Fwd	caccgTCCTTTATGGAGCAGCCGCT	
Skp2_2_Rev	aaacAGCGGCTGCTCCATAAAGGAc	
Cd8a_1_Fwd	caccGGTGTTGGGGTCCGTTTCGCA	*Cd8a* (mouse)
Cd8a_1_Rev	aaacTGCGAAACGGACCCCAACACC	
Cd8a_2_Fwd	caccGGCTGGGTGAGTCGATTATCC	
Cd8a_2_Rev	aaacGGATAATCGACTCACCCAGCC	
*SKP2*‐1‐Fwd	caccgTCCCTCCAAAGGTGTTTCAT	*SKP2* (human)
*SKP2*‐1‐Rev	aaacATGAAACACCTTTGGAGGGAc	
*SKP2*‐2‐Fwd	caccgCCAGAGACCTTTAGCAGCTC	
*SKP2*‐2‐Rev	aaacGAGCTGCTAAAGGTCTCTGGc	

### 2.2 Plasmids and virus transduction

For generation of lenti‐ or retroviral particles, 1.25 × 10^5^ HEK 293T cells were seeded per well in 24‐well plates. For transfection the plasmid of interest (400 ng), the viral packaging plasmid pSPAX2 (200 ng) for lentiviruses or HIT‐60 for retroviruses, and pVSVg (100 ng) for pseudotyping were used in 20 μL IMDM medium (Sigma‐Aldrich), mixed with 20 μL IMDM medium containing 1.2 μL polyethylenimine (PEI), incubated for 30 min at room temperature and added drop‐wise to the 293T cells. Virus particles were harvested after 40–48 h post transfection. Viral supernatants were mixed with polybrene (16 μg·mL^−1^ final concentration). Target cells were transduced using spin infections in 1.5 mL reaction tubes at 37 °C and 300 ***g*** for 90 min. After puromycin selection (2 μg·mL^−1^) for 2 days or cell sorting (via dsRed marker), 500 cells were seeded in 5 mL Baf3 medium containing 2% Methylcellulose and plated in a 10 cm bacterial plate. Single‐cell colonies were picked after 9–10 days.

### 2.3 CRISPR/Cas9 loss‐of‐function screen

Baf3 screen cells were generated by transduction with a construct encoding Cas9‐P2A‐Cd8 and sorted for CD8 expression. Virus production, infection of screen cells, gDNA isolation, PCR amplification, and bioinformatic analysis were performed as previously described [30]. Baf3 screen cells were treated with 300 nm PF‐477736 8 days after transduction. Treatment was washed out after 48 h and cells were recovered for 2–3 weeks. The control population was harvested after 8 days without treatment. Raw sgRNA reads can be found in Table S1.

### 2.4 TIDE PCR analysis

To identify SKP2 knock‐out clones, Tracking of Indels by Decomposition (TIDE) analysis [31] was performed according to the following protocol (https://doi.org/10.1093/nar/gku936), except that HOTSHOT DNA extraction was applied where cell pellets were resuspended in 30 μL Hotshot reagent (NaOH 25 mm, disodium EDTA 0.2 mm, pH = 12.0) for 30 min at 95 °C, then 30 μL neutralizing reagent (TRIS–HCl 40 mm) was added for 20 min on ice. A Monarch DNA Gel Extraction Kit for PCR product purification was used. To amplify the DNA, the following PCR program was used: DNA denaturation at 98 °C for 30 s, then DNA amplification using 35 repeats of 98 °C 20 s, 60 °C 20 s and 72 °C 45 s. After 35 cycles a final step of 72 °C for 3 min was performed (Table 2).

**Table 2:** Sequencing primers.

Primer name	Sequence 5′–3′
Skp2 SCC_1351F	agaatgtctgcccttctgca
Skp2 SCC_1351R	ccatttcactgccacacttcc
Skp2 SCC_1352F	aaggaagtccagtggccatg
Skp2 SCC_1352R	cccgaggaatcagacagcat

PCR products were sent to Sanger sequencing. Sanger sequencing data was uploaded to the TIDE analysis web tool (https://tide.deskgen.com) for analysis.

### 2.5 DNA‐content analysis by propidium‐iodide (PI)‐staining

Cells were fixed in 1 mL ice‐cold ethanol (70%) and stored at −20 °C for at least 20 min. Then cells were washed once with PBS (200 ***g*** for 5 min) and incubated with 250 μL PBS containing Propidium iodide (f.c. 40 μg·mL^−1^) and RNAseA (f.c. 100 ng·mL^−1^) at 37 °C for 30 min.

### 2.6 Phospho‐histone H3^Ser10^ analysis

Cells were fixed in 1 mL ice‐cold ethanol (70%) and stored at −20 °C for at least 20 min. After two washes with 2 mL PBS (1900 r.p.m. for 5 min), cells were incubated for 15 min in PBS containing Triton‐X‐100 (0.25%, Sigma) on ice for permeabilization. Cells were washed in 2 mL PBS containing 1% BSA and then incubated with the antibody solution (50 μL antibody solution/sample; antibody: alexa488‐phH3 (Ser28) 1 : 100, eBioscience) for 60 min at room temperature. Cells were washed in 2 mL PBS containing 1% BSA, followed by DNA staining using DAPI (250 ng·mL^−1^) in 300 μL PBS containing RNAseA (f.c. 100 ng·mL^−1^) at 37 °C for 30 min.

### 2.7 EdU labeling and detection

Baf3 cells were cultured in the presence of EdU (10 μm final concentration using the EdU Flow Cytometry Cell Proliferation Assay, Thermo Fisher Scientific, Vienna, Austria) for 1 h. Cells were washed two times with 2 mL PBS and then incubated for 20 min in 1% methanol‐free paraformaldehyde (Thermo Fisher Scientific) on 4 °C for fixation. Cells were washed in 2 mL PBS containing 1% BSA and permeabilized with a saponin‐based reagent (permeabilization buffer) for 15 min on room temperature. The labeling mix (200 μL per sample) was prepared as follows: 10 μL Tris (2 m, pH 8.5), 145 μL H_2_O, 4 μL CuSO_4_ (100 μm), 1 μL Cy‐azide (10 μm in DMSO) and 40 μL ascorbic acid (0.5 m). Cells were incubated with the labeling mix for 30 min at room temperature in the dark. Cells were washed in permeabilization buffer, followed by DNA staining using Hoechst 33342 (40 μm) in 100 μL permeabilization buffer for 10 min at room temperature. PBS was added and samples were measured using a flow cytometer.

### 2.8 Immunoblotting

Cells were lysed in 50 mm Tris pH 8.0, 150 mm NaCl, 0.5% NP‐40, 50 mm NaF, 1 mm Na_3_VO_4_, 1 mm PMSF, one tablet protease inhibitors (EDTA free, Roche) per 10 mL and 30 μg·mL^−1^ DNaseI (Sigma‐Aldrich) and analyzed by immunoblotting as described before [19, 24]. To load equal amounts of proteins on a 12% denaturing polyacrylamide gel a Bradford assay was performed. The gel was run at 100 V. The wet transfer on a nitrocellulose membrane was performed for 65 min at 100 V. The membrane was blocked with 5% milk in PBS‐T (0.1% Tween) for 1 h at room temperature and incubated overnight with the respective primary antibodies (5 mL per membrane):rabbit anti‐Bax (CS2772), rabbit anti‐Bak (CS3814), rabbit anti‐GAPDH (CS 2118, 14C10) 1 : 5000, mouse anti‐HSP90 (Santa Cruz Biotechnology, Inc., Heidelberg, Germany, Szabo‐Scandic HandelsgmbH, Vienna, Austria, 13119) 1 : 5000, rabbit anti‐SKP2 (Ag6826, Proteintech Germany GmbH, Planegg‐Martinsried, Germany, 15 010‐1‐AP) 1 : 1000, anti‐p27 (p27 clone 57, HRP‐conjugated p27 antibody, BD, Becton Dickinson Austria, Vienna, Austria) 1 : 10 000, mouse anti‐CYCLIN D3 (CS DCS22) 1 : 1000, rabbit anti‐CYCLIN E2 (CS 4132) 1 : 1000 and rabbit anti‐CYCLIN A2 (E67.1, Santa Cruz). HRP‐conjugated mouse anti‐p27^*Kip1*^ [32] (clone 57, BD Bioscience), 1 : 15 000. The membrane was then washed five times in PBS‐T at room temperature and incubated with the secondary antibody anti‐rabbit‐HRP (Sigma‐Aldrich Handels GmbH, Vienna, Austria, 1 : 10 000 in 5% milk PBS‐T) at room temperature for 1 h. The membrane was washed five times with PBS‐T and WesternBright ECL‐substrate spray (Advansta, San Jose, CA, USA (Distributor) Biozym Biotech Trading GmbH, Vienna, Austria, K‐12049‐D50) was added for signal detection.

### 2.9 Cell sorting and flow cytometry

Flow cytometric analysis was performed on an LSR Fortessa (BD) or ATTUNE NxT (Thermo Fisher Scientific), whereas cell sorting was performed on a FACS‐Aria‐III (BD, Beckton Dickinson, Vienna, Austria). FACS data were analyzed using flowjo v10.8 software. Hoxb8‐FLT3 cells transduced with a construct containing Cas9 and CD8 as a marker were sorted using CD8‐PE (BD, Clone 53–6.7), 1 : 200.

### 2.10 Statistical analysis

Statistical analysis of the genome‐wide CRISPR/Cas9 loss‐of‐function screen was included in the edger analysis tool [33]. Statistical analysis was performed using unpaired *t*‐tests and prism graphpad software 9.2.0 (GraphPad Software, San Diego, CA, USA). Experiments with the readout ‘fold enrichment’ cannot undergo statistical testing because virus production and infection were always performed fresh and varied in efficacy. This fact does not coincide with statistical variance of our biological phenotypes, but would falsify any type of statistical test we would perform. After consulting a statistician we agreed to supply the raw transduction efficacies (DMSO, CHK1i) in the Table S2.

## 3 Results

### 3.1 A CRISPR/Cas9‐based loss‐of‐function screen identifies potential regulators of CHK1i‐induced cell death

**Fig. 1:**
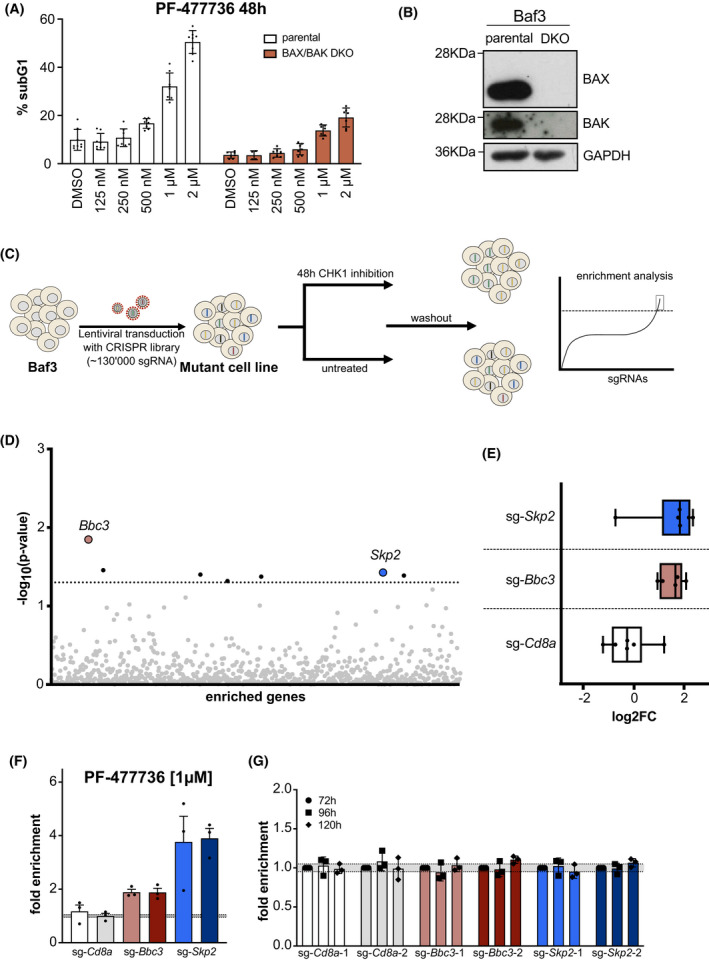
A CRISPR/Cas9 loss‐of‐function screen identifies SKP2 and PUMA/BBC3 as potential regulators of CHK1i‐induced cell death. (A) Parental and BAX/BAK double‐knockout (DKO) Baf3 cells were cultured for 48 h in different concentrations of the CHK1‐inhibitor, PF‐477736. Cell death was assessed using PI‐staining and flow cytometry. Each bar represents the mean of the respective concentration (±SD) of eight independent experiments (parental), seven independent experiments (BAX/BAK DKO; DMSO, 250 nm, 500 nm, 1 μm, and 2 μm) and four independent experiments for 125 nm. (B) Western blot confirming BAX/BAK double‐deficiency. A single experiment was performed. (C) Mutagenesis screen work‐flow: Murine Baf3^Cas9^ cells were transduced with the mouse GeCKO v2 library targeting each annotated gene with 4–6 individual sgRNAs. Upon *in vitro* expansion, cells were split and treated for 48 h with PF‐477726 or vehicle control (DMSO). After wash out, surviving cells were cultured for 2–3 weeks and genomic DNA was isolated for next‐generation Illumina sequencing. (D) NGS enrichment analysis. Each dot represents a single gene, which showed an enrichment in the library (either two or three sgRNAs) across all three independent biological replicates. The dotted line represents the significance level of 0.05. (E) Enrichment analysis of six independent sgRNAs targeting *Bbc3/Puma* and *Skp2* compared to the *Cd8a* controls from three independent experiments. Data is depicted as box plots. Whiskers mark the highest and the lowest enriched sgRNAs. (F) For validation, Baf3^Cas9^ cells were transduced with virus encoding the sgRNA of interest and a dsRed marker gene (sg‐GOI/dsRed). sgRNAs targeting the *Cd8a* gene, not expressed in Baf3 cells were used as controls. Three days post transduction cells were treated with the CHK1 inhibitor PF‐477736 [300 nm] or DMSO for 48 h. The percentage of dsRed^+^ cells was assessed using flow cytometry, whereby the treatments were normalized to the DMSO‐treated controls and are shown as the ratio between (%dsRed^+^_treatment_ to %dsRed^+^_DMSO_). Bars indicate the mean fold enrichment (±SEM) of three independent experiments. (G) Same procedure as in (F), but the sg‐GOI/dsRed transduced cells were monitored for enrichment over time without treatment. Bars indicate the mean fold enrichment (±SEM) of three independent experiments.

Our previous work demonstrated that pharmacological CHK1 inhibition promotes BCL2‐regulated apoptosis in multiple hematopoietic cell types [19, 24]. In order to identify new regulators of CHK1i‐induced cell death, we decided to conduct an unbiased genome‐wide CRISPR/Cas9‐based loss‐of‐function screen in murine Baf3 pro‐B cells that respond to CHK1‐inhibition by the initiation of BAX/BAK‐dependent apoptosis (Fig. 1A,B and Fig. S1A, Table 1). Therefore, we transduced Cas9‐expressing Baf3 cells with the lenti viral genome‐wide mouse GeCKO v2 CRISPR‐sgRNA library. Cells were treated for 48 h with the CHK1 inhibitor PF‐477736 followed by drug wash‐out or left untreated. Genomic DNA was extracted from surviving cells and subjected to next‐generation Illumina sequencing to identify enriched sgRNAs (Fig. 1C). Using a stringent cut‐off where half the number of the sgRNAs present in the library needed to show enrichment (i.e., 3/6 per gene) and across three independent biological replicates, four target genes (*Bbc3*, *Skp2*, *Stub1*, *mmu‐miR‐3082*) remained that, once lost, may provide a survival advantage to Baf3 cells in the presence of CHK1i. Next to the BH3‐only protein and p53 target, *Bbc3/Puma*, previously implicated in cell death upon CHK1i [24], the S‐phase kinase‐associated protein 2 (SKP2), a potentially new player in cell death regulation induced by CHK1‐inhibition, showed the most significant *P*‐value (Fig. 1D) and the highest ‘fold‐change’ in transcript‐enrichment when compared to non‐targeting guide RNAs (Fig. 1E; Table S1).

To rule out off‐target effects among the top hits, we established a validation protocol using two structurally different CHK1 inhibitors, CHIR‐124 and PF‐477736, in a pre‐titrated dose that resulted in 30–40% cell death after 48 h of treatment (Fig. 1A and Fig. S1A). To track transduced cells by flow cytometry we used lentiviral plasmids encoding for the sgRNA of interest and a dsRed marker gene (sg‐GOI/dsRed). We decided to validate *Bbc3/Puma* and *Skp2* from the candidate list using two individual sgRNAs found to be enriched in our screen and compared them to two control sgRNAs targeting the T cell surface marker *Cd8a*, not expressed in Baf3 cells (sg‐Cd8a/dsRed). Three days after transduction cells were treated with the two different CHK1i for an additional 48 h and monitored for their survival. To unify the data and account for differences in transduction efficiency between experiments (Table S2), we normalized the results received in flow cytometry by dividing the percentage of surviving dsRed^+^ cells of CHK1i‐treated cells by that found in DMSO‐treated cells (% dsRed^+^_treatment_/% dsRed^+^_DMSO_). This results in a numerical increase of dsRed^+^ cells > 1 whenever loss of the targeted gene provides protection from apoptosis, displayed as ‘fold enrichment’ while a sensitization to cell death would be indicated by mean values < 1. Cells transduced with sg‐*Bbc3*/dsRed constructs showed a 1.5‐ to 2‐fold enrichment when treated with CHK1i in comparison to DMSO controls. No such increase was observed when cells were transduced with the non‐targeting sg‐*Cd8a*/dsRed constructs. This result is in line with our previous findings that PUMA‐deficient hematopoietic progenitor cells are partially protected from CHK1i treatment [24]. However, cells transduced with the sg‐*Skp2*/dsRed constructs showed a much stronger enrichment of dsRed^+^ cells after treatment (Fig. 1F and Fig. S1B). In order to rule out that the observed effects were due to differences in basal proliferation rates upon loss of SKP2, we monitored the percentage of dsRed^+^ cells over time without treatment. Of note, the percentage of dsRed^+^ cells did not change significantly over an observation period of 5 days, indicating that the observed effect cannot simply be explained by an altered proliferation rate in cells expressing guide RNAs targeting *Bbc3/Puma* or *Skp2* (Fig. 1G).

To exclude that the survival advantage that we observed upon loss of SKP2 in Baf3 cells upon CHK1‐inhibition may be cell line or cell type specific, we also tested Hoxb8‐immortalized murine multi‐potent progenitor‐like cells, i.e., Hoxb8^FLT3^ cells, that we showed previously to be partially protected from CHK1i treatment when lacking the BH3‐only protein PUMA/BBC3 [24]. Comforting enough, we again observed a significant accumulation of dsRed^+^ cells in response to CHK1i treatment when using sgRNAs targeting *Skp2*, confirming the phenomenon also in another murine hematopoietic cell line (Fig. S1C).

Together, these findings confirm that BCL2‐regulated cell death is critical for the proapoptotic effects of CHK1 inhibition. However, a link between SKP2 expression levels and CHK1i‐induced cell death has not yet been reported. The established role of *SKP2* as a putative oncogene and its key role in cell cycle regulation prompted us to interrogate its potentially pro‐apoptotic effects in CHK1i‐treated cells in more detail.

### 3.2 The F‐box protein SKP2 defines cellular sensitivity to CHK1 inhibition

**Fig. 2:**
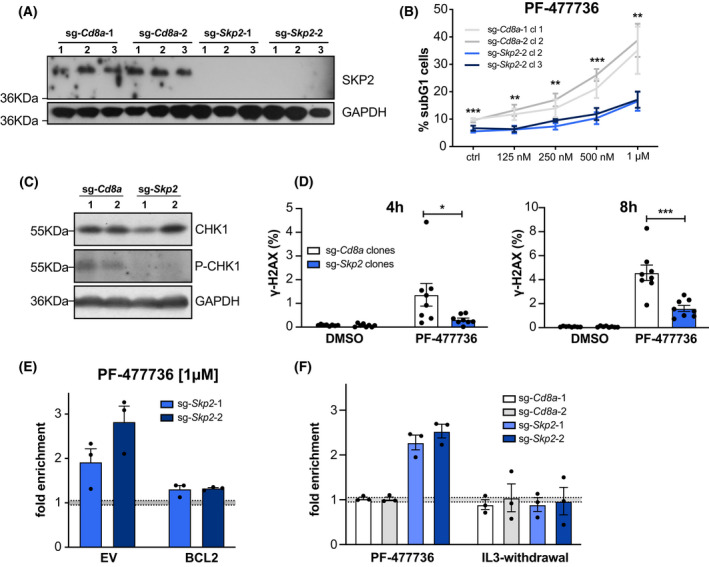
The F‐box protein SKP2 defines cellular sensitivity to CHK1 inhibition. (A) Western blot analysis confirming loss of protein in Baf3 SKP2 KO clones. A single experiment was performed (B) Two randomly selected clones of each genotype were cultured for 48 h in different doses of CHK1‐inhibitor PF‐477736. Cell death was assessed using PI‐staining and flow cytometry. Data points indicate means ± SEM of three independent experiments. (C) Western blot assessing CHK1 and pCHK1^Ser345^ in SKP2 KO and sg‐*Cd8a* control clones. (D) Baf3 SKP2 KO‐ and sg‐*Cd8*a control clones were treated for 4 or 8 h with 1 μm PF‐477736. DNA damage was assessed by γ‐H2A.X^Ser139^ and DAPI staining using flow cytometry. Bars indicate the mean percentage (±SD) of four independent experiments in two clones analyzed per genotype. (E) Baf3 cells expressing pMIP‐BCL2 or an empty vector (EV) control were transduced with two independent sg‐*Skp2*/dsRed or sg‐*Cd8a*/dsRed constructs. Three days post transduction the respective bulks were treated with the CHK1 inhibitor PF‐477736 [1 μm] or DMSO for 48 h. The percentage of dsRed^+^ cells was assessed using flow cytometry, whereby the treatments were normalized to the DMSO‐treated controls. Bars indicate the mean fold enrichment (±SEM) in three independent experiments. (F) Baf3 cells were transduced with two independent sg‐*Skp2*/dsRed or sg‐*Cd8a*/dsRed constructs. Three days post transduction the respective bulk cultures were split and treated either with the CHK1 inhibitor PF‐477736 [1 μm] or DMSO for 48 h (left panel); or depleted for IL‐3 or left untreated for 48 h (right panel). The percentage of dsRed^+^ cells was assessed using flow cytometry, whereby the PF‐477736‐treated cells were normalized to the DMSO‐treated controls, and the IL‐3‐depleted cells were normalized to the untreated controls. Bars indicate the mean fold enrichment (±SEM) noted in three independent experiments. *P < 0.05; **P < 0.01; ***P < 0.001; unpaired *t*‐test.

To further validate the beneficial effect of *Skp2*‐deficiency, we generated individual knock‐out clones using two different sgRNAs (Fig. 2A) and assessed their response to CHK1‐inhibition. In line with the above, *Skp2*‐deficient Baf3 cells died substantially less upon CHK1‐inhibition when compared to *Cd8a*‐CRISPR control cells (Fig. 2B). Interestingly, *Skp2*‐deficient Baf3 cells also represented with a lower basal CHK1‐activity in steady state, as indicated by low to undetectable levels of Ser^345^ phosphorylation of CHK1. However, no changes in total CHK1 protein levels were observed (Fig. 2C). This observation indicated that *Skp2*‐KO cells may accumulate less DNA damage in steady state that triggers CHK1 activation during DNA replication in S‐phase. To assess this further, we treated *Cd8a* control and S*kp2*‐deficient cells with the CHK1i‐inhibitor PF‐477736 and analyzed the levels of phosphorylated γH2A.X^Ser139^, a well‐established marker of DNA damage by flow cytometry. Remarkably, *Skp2*‐deficient Baf3 cells treated with inhibitor showed clearly reduced levels of DNA damage (Fig. 2D). Together, this suggested that *Skp2*‐deficient cells may die less in response to CHK1‐inhibition because they accumulate less DNA damage, a notion consistent also with the involvement of PUMA in executing DNA damage‐induced apoptosis [26, 27].

Next, we asked whether impaired apoptosis initiation is indeed solely responsible for the accumulation of *Skp2*‐deficient cells in response to CHK1‐inhibition. To answer this question, we assessed whether deletion of *Skp2* is still beneficial when mitochondrial apoptosis was generally impaired. To do so, we generated Baf3 cells that stably overexpress anti‐apoptotic BCL2 or an empty vector (EV) and transduced those cells with sgRNAs targeting *Skp2*. Strikingly, *Skp2*‐deficiency did no longer cause an accumulation of cells after inhibitor treatment on a BCL2‐transgenic background (Fig. 2E and Fig. S1D), demonstrating that cells lacking SKP2 expression indeed benefit from reduced apoptosis in response to CHK1i. Of note, *Skp2* deletion did not confer cell death resistance *per se*, as apoptosis of Baf3 cells induced by IL‐3 deprivation was unchanged (Fig. 2F).

**Fig. 3:**
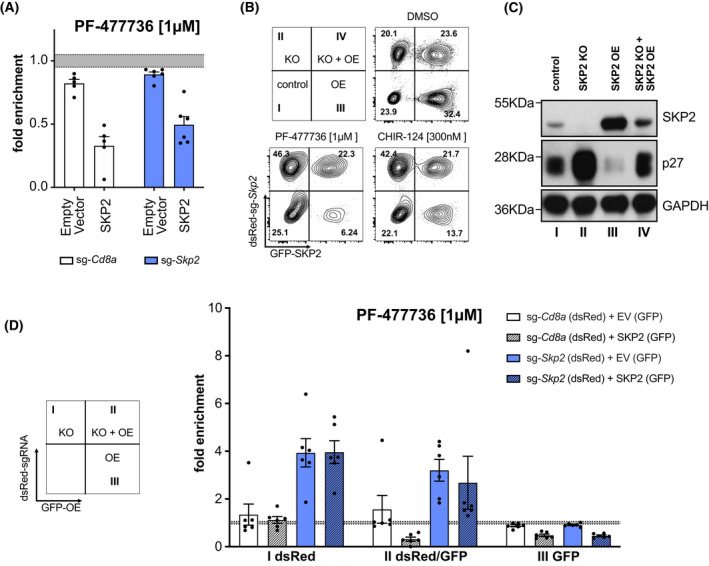
Exogenous SKP2 restores sensitivity to CHK1 inhibitors. (A) Baf3 SKP2 KO and sg*Cd8a* control clones (dsRed^+^) were transduced with human SKP2 or an empty vector (EV), which both express GFP as a marker. Three days post transduction cultures were treated with the CHK1i PF‐477736 [1 μm] or DMSO for 48 h. The percentage of GFP^+^dsRed^+^ cells was assessed using flow cytometry. Treatments were normalized to the DMSO‐treated controls. Bars indicate the mean fold enrichment (±SEM) of two individual clones per genotype noted in three independent experiments (Cd8a‐1, Skp2‐1, Skp2‐2) and two independent experiments (Cd8a‐2). (B) Representative contour plots of parental cells mixed with SKP2 KO for rescue experiment, depicting the distribution of the four subpopulations (I–IV), with and without treatment of CHK1i after 48 h. Three independent experiments were performed (C) Western blot showing SKP2 and p27 levels of the respective, sorted subpopulations shown in C (DMSO treatment). A single experiment was performed. (D) Quantification of sg‐*Skp2*/dsRed or sg‐*Cd8a*/dsRed transduced cells reconstituted with human SKP2 (GFP) or EV (GFP) after 48 h of treatment. The PF‐477736 treated cells were normalized to the DMSO‐treated cells. Bars indicate the mean fold enrichment (±SEM) of two independent sgRNAs (targeting *Cd8a* or *Skp2*) in three independent experiments.

Next, we performed rescue experiments by reconstituting *Skp2* knockout cells with human *SKP2*, inert to the selected mouse‐specific sgRNAs. Toward this end, we transduced sg*‐Skp2/dsRed* and sg*‐Cd8a/dsRed* expressing Baf3 clones retrovirally with a construct encoding human SKP2‐IRES‐GFP (SKP2), or with an empty vector control IRES‐GFP (EV). Three days after transduction, cells were treated for 48 h with CHK1i, as described above. Strikingly, we could observe that cells expressing exogenous human SKP2 became again more sensitive to CHK1i treatment, as the percentage of dsRed^+^/GFP^+^ double‐positive cells expressing human SKP2 strongly declined, when compared to the dsRed^+^ single‐positive cells lacking mouse SKP2 (Fig. 3A and Fig. S2A). Of note, the same actually held true when sg‐*Cd8a*/dsRed control cells were transduced with human SKP2 alone, indicating that overexpression of SKP2 *per se* rendered these cells more susceptible to CHK1‐inhibition. Importantly, we did not observe a reduction of cells that were transduced with empty vector controls (Fig. 3A and Fig. S2A).

To further validate the impact of SKP2 protein levels on CHK1‐inhibitor susceptibility, SKP2 KO clones (dsRed^+^) were mixed with Baf3 Cas9 cells without reporter and transduced in bulk with SKP2‐IRES‐GFP to assess the susceptibility to CHK1 inhibition in comparison to parental cells. Like this, we generated four different traceable subpopulations of cells within each sample. Cells were either wild‐type for *Skp2* (no color), *Skp2*‐deficient (dsRed^+^), *SKP2*‐overexpressing (GFP^+^) or deficient for mouse SKP2, re‐expressing human SKP2 protein (dsRed^+^/GFP^+^). Cells deficient for SKP2 did accumulate (quadrant II) after CHK1 inhibition whereas SKP2 overexpressing cells (quadrant III) were more sensitive to CHK1 inhibition. Importantly, reconstituted cells, i.e., SKP2 KO cells expressing human SKP2 (quadrant IV) were seemingly unaffected by CHK1i. DMSO‐treated cells were sorted to analyze the protein levels of the four sub‐populations by western analysis (Fig. 3B,C).

To exclude adaptation processes in single cell‐derived clones in culture, we aimed to acutely deplete mouse SKP2 and express the human SKP2 rescue construct simultaneously. To do so, we transduced Baf3^Cas9^ cells with sg‐*Skp2*/dsRed or sg‐*Cd8a*/dsRed control constructs and 2 days later transduced these bulks again with the CRISPR‐resistant version of human SKP2 (SKP2‐IRES‐GFP) or the empty vector control (Fig. 3D). Three days after the second transduction, cells were treated with DMSO or CHK1i for 48 h prior flow cytometric analysis. As expected, neither transduction with the control sgRNA targeting *Cd8a*, nor transduction with the empty control vector, alone or in combination, affected the GFP/dsRed distribution in response to CHK1 inhibition. Furthermore, overexpression of human SKP2 rendered these cells again more susceptible to CHK1i‐inhibition (Fig. 3D and Fig. S2B). Strikingly, while deletion of endogenous *Skp2* conferred again drug‐resistance, as seen before in single‐cell clones, simultaneous rescue by human SKP2 overexpression neutralized the protective effect of *Skp2*‐deletion. In contrast, overexpression of SKP2 alone reduced cellular fitness further (Fig. 3D and Fig. S2C).

In summary, SKP2 protein levels define the susceptibility of Baf3 and HoxB8^FLT3^ cells to CHK1i‐induced cell death. We hence hypothesized that changes in p27 protein levels might phenocopy the observed effects if it was the key substrate of SKP2 in this setting.

### 3.3 The SKP2‐p27 axis defines cell death susceptibility upon CHK1 inhibition

**Fig. 4:**
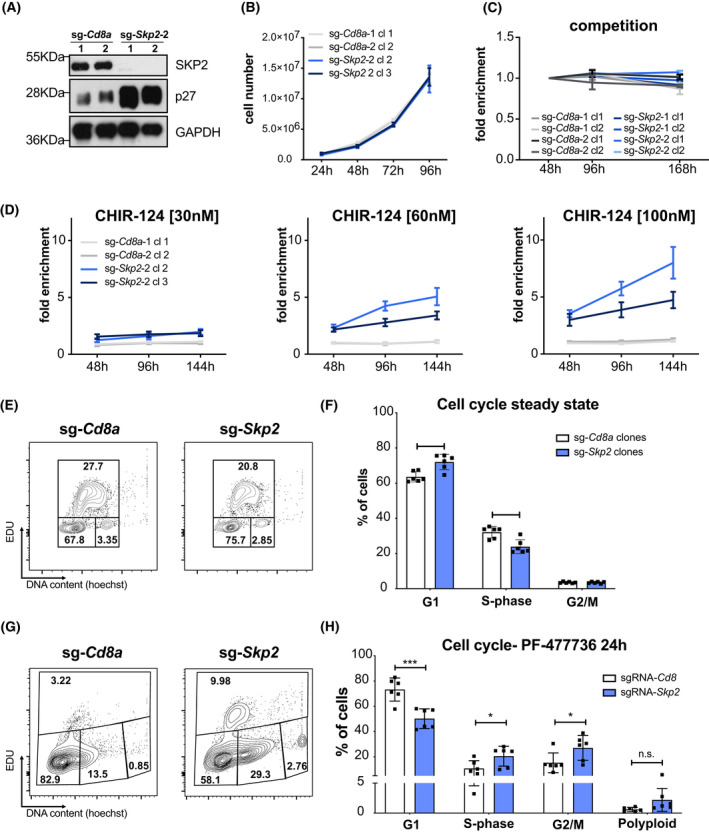
Accumulation of p27 provides a survival advantage in the presence of CHK1i. (A) Western blot showing SKP2 and p27 protein levels in SKP2 KO and control clones. A single experiment was performed. (B) Baf3 SKP2 KO and sg‐*Cd8a* control clones were seeded at a concentration of 3.5 × 10^5^ cells per well. Cells were split 1 : 1 and counted every 24 h over the course of 4 days using an Attune NxT Flow cytometer. Line graph data points represent the mean (±SD) of three technical replicates per time point and three individual experiments per clone. (C) Baf3 SKP2 KO and sg‐*Cd8a* control clones (dsRed^+^) were mixed 1 : 1 ratio with Baf3^Cas9^ cells (parental cells) and the percentage of dsRed^+^ cells was monitored over time using flow cytometry. Each line represents the mean (±SD) of dsRed^+^ (%) cells normalized to the 48 h timepoint in three independent experiments per clone, except sg‐Cd8a‐1cl1 and sg‐Cd8a‐2cl2 (*n* = 2). (D) Baf3 SKP2 KO and sg‐*Cd8a* control clones (dsRed^+^) were mixed at a ratio of 15/85 with parental Baf3^Cas9^ cells and treated with low doses of the CHK1 inhibitor CHIR‐124 for up to 7 days. The percentage of dsRed^+^ cells was assessed using flow cytometry, whereby the time points were normalized to 0 h. Cells were split every 2–3 days. Lines represent mean ratio (±SEM) of three independent experiments. (E) Representative contour plots of EdU cell cycle analysis of a Baf3 SKP2 KO and sg‐*Cd8a* control clone. Three independent experiments were performed. (F) Quantification of cell cycle analysis of Baf3 SKP2 KO and sg‐*Cd8a* control clones using EdU‐staining. Bars indicate the mean percentage (±SD) of two independent single‐cell clones per genotype analyzed in three independent experiments. (G) Representative contour plots of EdU cell cycle analysis of Baf3 SKP2 KO and sg‐*Cd8a* control clones after 24 h of PF‐477736 treatment. Three independent experiments were performed. (H) Quantification of cell cycle analysis of Baf3 SKP2 KO and sg‐*Cd8a* control clones using EdU‐staining after 24 h of PF‐477736 treatment. Bars indicate the mean percentage (±SD) of two different single‐cell clones per genotype analyzed in three independent experiments. *P < 0.05; **P < 0.01; ***P < 0.001; unpaired *t*‐test.

Western blot analysis and TIDE PCR sequencing (Table 2) demonstrated that the single‐cell clones showed complete loss of SKP2 protein and a reciprocal increase in p27 levels (Fig. 4A), as noted before in our transient assays. To characterize these cells further, we conducted again cell proliferation assays. Interestingly, despite the high p27‐protein levels in SKP2‐deficient Baf3 cells their proliferation rate was comparable to control cells (Fig. 4B). Furthermore, SKP2‐deficient single‐cell clones did not outcompete parental cells or *vice‐versa* in mixed cultures over an observation period of 7 days (Fig. 4C), confirming that there was no proliferation defect that may reduce sensitivity to CHK1i. Yet, when we mixed *Skp2*‐KO cells with parental cells at a 15/85% ratio, we observed that *Skp2*‐KO cells were able to outcompete parental cells in response to CHK1i in a dose and time‐dependent manner. This was not seen when using sg‐*Cd8a* control cells (Fig. 4D).

Assessing the different cell cycle phases using an EdU incorporation assay (Fig. 4E), we observed that *Skp2*‐deficient Baf3 cells show a significant increase of cells in G1‐ and a drop of cells in S‐phase, a finding expected from cells having elevated p27 protein levels. Percentages of cells in G2/M remained unchanged (Fig. 4F). Upon CHK1i treatment control cells showed a clear drop in S‐phase and a relative increase in the other cell cycle phases, suggesting that apoptosis is mainly happening in S‐phase (Fig. 4F,H). In the absence of SKP2, however, the fraction of S‐phase cells remained largely unchanged, indicating improved survival, while cells accumulated in G2/M as well as in a polyploid state, albeit statistical significance was not reached in the latter (Fig. 4F,H).

Taken together, these observations suggest that CHK1 inhibition triggers cell death mainly in S‐phase and promotes cytokinesis failure. Our findings also suggest that p27 exerts an anti‐apoptotic function in this setting, or that SKP2 acts independently of p27 to promote cell death upon CHK1 inhibition.

### 3.4 p27 determines sensitivity to CHK1i in the absence of SKP2

**Fig. 5:**
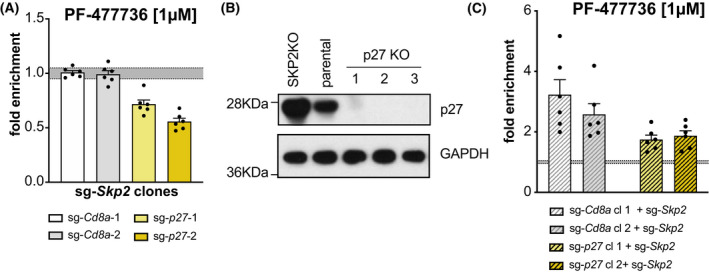
p27 is the key SKP2 substrate that defines CHK1i sensitivity. (A) Baf3 SKP2 KO clones were transduced with two independent sg‐*p27*/dsRed or sg‐*Cd8a*/dsRed constructs, respectively. Three days post transduction the bulks were treated with PF‐477736 [1 μm] or DMSO for 48 h. The percentage of dsRed^+^ cells was assessed using flow cytometry, whereby treated cells were normalized to DMSO controls. Bars indicate the mean percentage (±SEM) of two independent clones analyzed in three independent experiments. (B) Western blot showing p27 levels in Baf3 p27 KO clones, Skp2 KO, and parental Baf3 cells. (C) Baf3 p27‐KO and sg‐*Cd8a* control clones were transduced with two different sg‐*Skp2*/dsRed constructs. Three days post transduction the cells were treated with CHK1 inhibitor PF‐477736 [1 μm] for 48 h. The percentage of dsRed^+^ cells was assessed using flow cytometry, whereby treated cells were normalized to DMSO controls. Bars indicate the mean percentage (±SEM) of two independent clones per genotype in three independent experiments.

We wondered whether p27 deletion can abrogate the effect of SKP2 loss, meaning that *Skp2/p27* double‐deficient cells would no longer show improved survival after exposure to CHK1i when compared to *Skp2*‐KO cells. Hence, *Skp2*‐deficient Baf3 cells that were generated with sgRNA‐puro constructs were transduced with sgRNAs targeting *p27* (sg‐*p27*/dsRed) and treated with CHK1i, as described above. Interestingly, the percentage of *Skp2/p27* double‐deficient cells (yellow bars) significantly declined in response to CHK1 inhibitor treatment as compared to the non‐transduced cells lacking only *Skp2*. Transduction with the control sgRNA targeting CD8a (white and gray bars) did not cause such an effect (Fig. 5A and Fig. S3A). Vice versa, when we transduced Cd8a‐ or p27‐KO clones with sgRNAs targeting *Skp2* (sgRNA‐*Skp2*/dsRed), the survival advantage was much more pronounced in the *Cd8a*‐control clones as compared to the p27‐KO clones (Fig. 5C and Fig. S3B).

Together, this indicates that the levels of p27 are indeed decisive when cells are treated with CHK1‐inhibitors, presumably by slowing down G1/S phase entry and/or progression. Hence, we conclude that the effect noted in *Skp2*‐deficient Baf3 cells indeed is due to the p27 protein accumulation and, presumably, CDK‐Cyclin complex inhibition.

### 3.5 SKP2 expression tunes drug sensitivity to CHK1i treatment in human pre‐B ALL cells

**Fig. 6:**
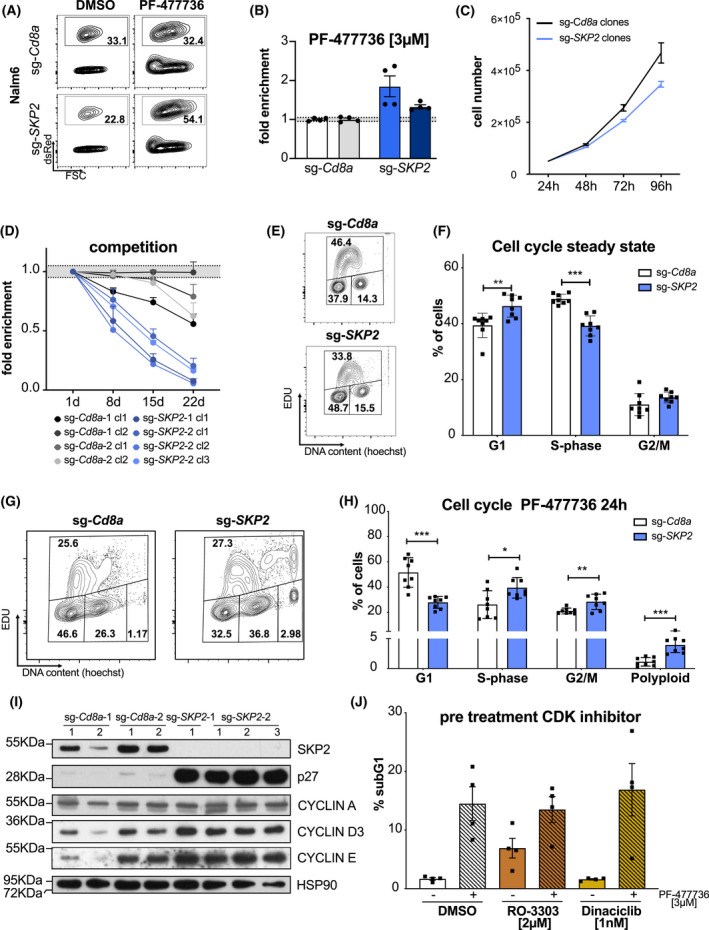
SKP2 expression tunes drug sensitivity to CHK1i in human cancer cells. Representative contour plots (A) and (B) quantification of Nalm6 human pre‐B acute lymphoblastic lymphoma cells transduced with two individual sg‐*SKP2*/dsRed or sg‐*Cd8a*/dsRed constructs, respectively. Three days post transduction the bulk cultures were treated with PF‐477736 [3 μm] or DMSO for 48 h. The percentage of dsRed^+^ cells was assessed using flow cytometry, whereby treated cells were normalized to DMSO controls. Bars indicate the mean percentage (±SEM) of four independent experiments. (C) Nalm6 SKP2 KO and sg‐*Cd8a* control clones were seeded at a concentration of 4 × 10^4^ cells per well. Cells were split 1 : 1 and counted every 24 h for a period of 4 days (±SEM). Experiment was performed using four independent clones. (D) Nalm6 SKP2‐KO (dsRed^+^) and sg‐*Cd8a* control clones (dsRed^+^) were mixed 1 : 1 ratio with Baf3Cas9 cells (parental cells, GFP^+^) and the percentage of dsRed^+^ and GFP^+^ cells was monitored over time using flow cytometry. Percentage of dsRed^+^ was normalized to percentage of dsRed^+^ on day 1. Points indicate the mean (±SEM) of three independent experiments per clone. (E) Representative contour plots of the cell cycle distribution (G1‐phase, S‐phase, G2/M) of Nalm6 SKP2 KO and sg‐*Cd8a* control clones in steady state were assessed via EdU incorporation for 1 h and subsequent staining, followed by flow cytometric analysis (EdU/DNA). Two independent experiments were performed. (F) Quantification of cell cycle profiles of Nalm6 SKP2‐KO and sg‐*Cd8a* control clones as shown in E. Bars indicate mean percentage (±SD) of four independent clones per genotype analyzed in two independent experiments. (G) Representative contour plots of the cell cycle distribution (G1‐phase, S‐phase, G2/M, polyploid) of Nalm6 SKP2 KO and sg‐*Cd8a* control clones after 24 h PF‐477736 [3 μm] treatment were assessed via EdU incorporation for 1 h and subsequent staining, followed by flow cytometry (EdU/DNA). Two independent experiments were performed. (H) Quantification of cell cycle profiles of PF‐477736 treated Nalm6 SKP2‐KO and sg‐Cd8a control clones as shown in G. Bars indicate the mean percentage (±SD) of four independent clones per genotype analyzed in two independent experiments. (I) Western blot showing SKP2, p27, CYCLIN A, CYCLIN D3, and CYCLIN E protein levels in Nalm6 SKP2‐KO and sg‐Cd8A control clones. A single experiment was performed. (J) Nalm6 parental cells were pre‐treated with RO‐3303, Dinaciclib, or DMSO for 8 h and then subsequently treated with PF‐477736 [3 μm] or DMSO plus RO‐3303, Dinaciclib, or DMSO for another 24 h. Cell death was assessed using PI staining and flow cytometry. Bars indicate the mean percentage (±SD) of four independent experiments. *P < 0.05; **P < 0.01; ***P < 0.001; unpaired *t*‐test.

Last, we aimed to prove that the SKP2‐p27 axis also regulates the sensitivity of human cancer cells to CHK1‐induced apoptosis. To this end, we transduced Nalm6 human pre‐B acute lymphoblastic lymphoma cells with sgRNAs targeting human *SKP2* or *Cd8a*, respectively. Strikingly, we could recapitulate the findings made in HoxB8^FLT3^ and Baf3 cells. Also Nalm6 cells expressing the human SKP2‐specific sgRNAs accumulated over time in the presence of CHK1i (Fig. 6A,B). However, in contrast to murine Baf3 cells, Nalm6 cells lacking SKP2 showed reduced proliferation rates (Fig. 6C) and were outcompeted over time by SKP2 proficient cells in mixed cultures (Fig. 6D). Cell cycle profiling using EdU incorporation revealed again a reduced fraction of cells in S‐phase with a concomitant increase in the other cell cycle phases in the absence of SKP2 (Fig. 6E,F). Upon treatment with CHK1i, SKP2‐deficient cells showed a clear reduction in S‐phase cells, accompanied by a significant increase of cells in G2/M as well as polyploid cells, indicating again cell death in S‐phase and/or after failed cytokinesis is prevented by increased p27 levels (Fig. 6G,H). SubG1 analysis confirmed the strong induction of apoptosis in control cells treated with CHK1i (Fig. S3C,D). Western blot analysis confirmed deletion of SKP2 in Nalm6 cells and the expected accumulation of p27, along with a modest increase in its targets, CYCLIN D and E (Fig. 6I). To test if p27 may reduce cell death by affecting CDK2 activity in S‐phase, preventing replication catastrophe [34, 35], we tested if pretreatment of cells with the CDK2 inhibitor Dinaciclib would reduce cell death rates. RO‐3303, blocking mainly CDK1 and G2/M entry, was used as a control. Predetermined sublethal doses of these inhibitors failed to reduce CHK1i‐induced cell death (Fig. 6J and Fig. S3E). Taken together, these experiments show that CHK1i kills Nalm6 cells mainly in S‐phase and after failed cytokinesis and that increased p27 levels most likely reduce this cell death independently of its ability to block CYCLIN E.

## 4 Discussion

Starting from a CRISPR/Cas9‐based loss‐of‐function screen, we observed that SKP2 and p27 protein levels determine susceptibility to CHK1‐inhibition in different hematopoietic model cell lines. As expected, loss of SKP2 resulted in stabilization of p27, its main substrate for ubiquitination and subsequent proteasomal degradation [28, 29]. Consequently, SKP2‐deficient Baf3 cells showed reduced S‐phase activity in steady state. Surprisingly, this did not affect cell proliferation rates, as we could demonstrate that cell‐doubling times were comparable to those of parental or control guide RNA‐edited cells. As a consequence, *Skp2*‐deficient cells were also not displaced by control cells over time in the absence of CHK1i. In contrast, SKP2‐deficient Nalm6 cells that overexpressed p27 showed again a drop in S‐phase cells but were proliferating slower and were indeed outcompeted over time by control guide RNA‐edited cells in mixed cultures. Why the high levels of p27 in Baf3 cells did not impact on the overall proliferation rates may be explained by an extension of time spent in S‐phase in favor of shortened gap‐phases, but this was not experimentally tested. Moreover, a large fraction of the accumulating p27 protein may actually not be inhibitory as it may either be modified by IL‐3 driven tyrosine kinase‐mediated phosphorylation [36, 37], or it may simply be in vast excess over the CDK/Cyclin complexes it could interact with. Regardless, reduced S‐phase activity was associated with reduced DNA damage upon CHK1 inhibition and lower cell death rates in SKP2 KO cells which were seen in both cell types. This suggests that increasing S‐phase speed or rate of G1/S‐phase entry may sensitize to CHK1 inhibitors. Consistently, CHK1 inhibitors have proven highly effective toward rapidly cycling AML [18], Burkitt and DLBCL cells [19]. However, pretreating cells with a CDK2 inhibitor to slow down S‐phase failed to provide a significant survival advantage to wild‐type cells treated subsequently with CHK1i. Even though not extensively tested, together with the observation that Baf3 cells proliferate equally fast in the presence or absence of SKP2, we conclude that the inhibitory effects of p27 are not exerted via inhibition of CDK2/CYCLIN E complexes, previously implicated in tuning sensitivity to CHK1i by affecting MRE11 nuclease activity [34, 35].

Clearly, CHK1i treatment led to a rapid increase in DNA damage (this study and [19]), which translates into p53 activation and explains the enrichment of sgRNAs targeting *Puma/Bbc3* in our screen. PUMA/BBC3 is a p53 target gene [38, 39] and CHK1i‐induced DNA damage stabilizes p53 [19]. While loss of p53 does not suffice to rescue cells from the consequences of CHK1 deficiency [3] or chemical inhibition [19], PUMA, together with other proapoptotic BCL2 family proteins, in particular BIM and NOXA, was shown to promote cell death in this setting [24]. Of note, BIM can be engaged in a p53‐independent manner in response to DNA damage [40]. Hence, the reduced DNA damage noted in SKP2‐deficient cells may be a simple explanation for the reduced cell death noted upon CHK1i treatment targeting mainly S‐phase cells. Consistently, loss of *Skp2* was no longer protective on a BCL2 transgenic or on a *p27*‐deficient background. Loss of SKP2 did not affect cell death upon IL‐3 growth factor deprivation. This suggests that SKP2 acts pro‐apoptotic by controlling p27 levels specifically in the context of CHK1i by reducing S‐phase activity, but has no role in cell death induced by cytokine withdrawal.

Yet, it remains open for discussion if increased p27 levels may inhibit cell death by alternative means. Non‐canonical roles of p27 in the cytoplasm related to cell migration have been reported, e.g., via interaction with Rho proteins, but these effects most likely do not apply here [41, 42]. However, p27 was shown to be cleaved by apoptotic caspases and one may speculate that increased p27 levels may quench effector caspase‐activity during CHK1i‐induced apoptosis [43]. Mutation of endogenous D176, recognized and cleaved by caspase‐3 in p27, will help to address this question in future studies.

## 5 Conclusion

Overall, our experiments suggest that the noted effect upon SKP2 loss on CHK1 inhibitor sensitivity is mediated by increased p27 levels, leading to reduced S‐phase entry or progression via interaction with D‐type, E‐type and potentially also A‐type CYCLIN/CDK complexes [36, 44, 45]. SKP2 overexpression has been associated with poor patient survival and drug resistance in a variety of human cancers and its reported oncogenic properties appear to be linked mainly to reduced p27 levels allowing increased tumor cell proliferation [46, 47]. Our findings suggest that the relative expression levels of SKP2 and/or p27 can be linked to a therapeutic vulnerability and be exploited to stratify cancer patients considered for treatment with CHK1i.

## Conflict of interest

The authors declare no conflict of interest.

## Author contributions

ML and BFR conducted and designed experiments, analyzed data, prepared figures, wrote manuscript. MS generated BAX/BAK DKO cells. FP conducted experiments. TGS conducted bioinformatics analysis. SH established and designed the CRISPR‐screen. FS and AV designed research, analyzed data, wrote manuscript, and conceived the study.

### Peer review

The peer review history for this article is available at https://publons.com/publon/10.1002/1878-0261.13264.

## Supporting information


**Fig. S1.** Identification of SKP2 as a modulator of CHK1i‐induced apoptosis.

**Fig. S2.** Exogenous SKP2 restores drug‐responsiveness.

**Fig. S3.** p27 is the key SKP2 substrate that defines CHK1i sensitivity.

**Table S1.** Raw sgRNA reads of CRISPR/cas9 screen.

**Table S2.** Raw data of viral transduction efficacies.

## Data Availability

Raw sgRNA counts from the GeCKO screen can be found in Table S1; flow cytometry data recording viral transduction efficiencies can be found in Table S2; all other data that support the findings of this study are available from the corresponding author upon reasonable request.
