# Supplementary figures and images for: The SKP2‐p27 axis defines susceptibility to cell death upon CHK1 inhibition
Source: Mol Oncol. 2022 Jul 7;16(15):2771–87. doi: 10.1002/1878-0261.13264 (PMC9348596; doi:10.1002/1878-0261.13264)

**A**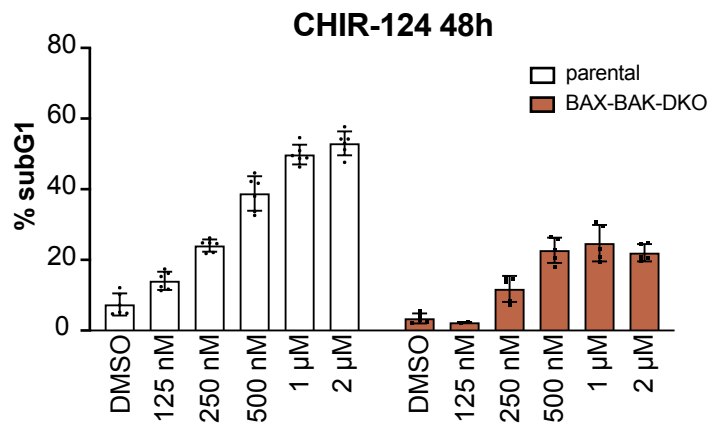**B**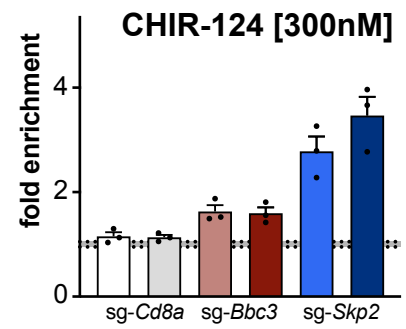**C**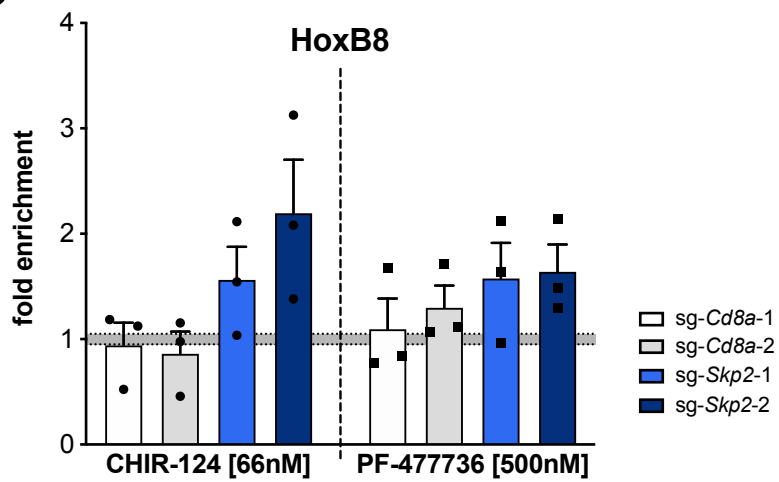**D**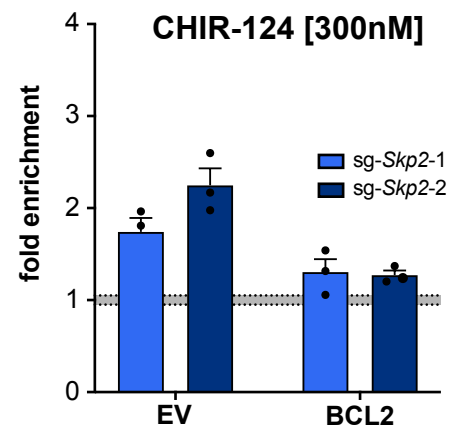

Supplement: Supplementary file 1 — Fig. S1. Identification of SKP2 as a modulator of CHK1i‐induced apoptosis. [file MOL2-16-2771-s006.pdf]

**A**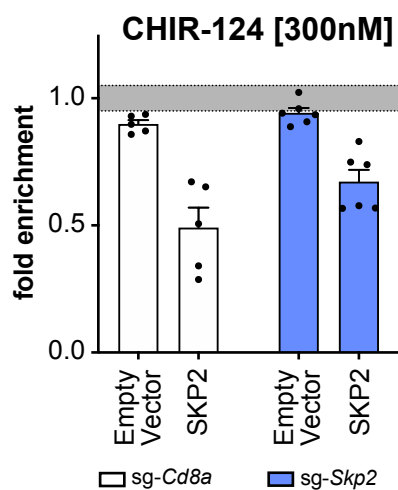**B**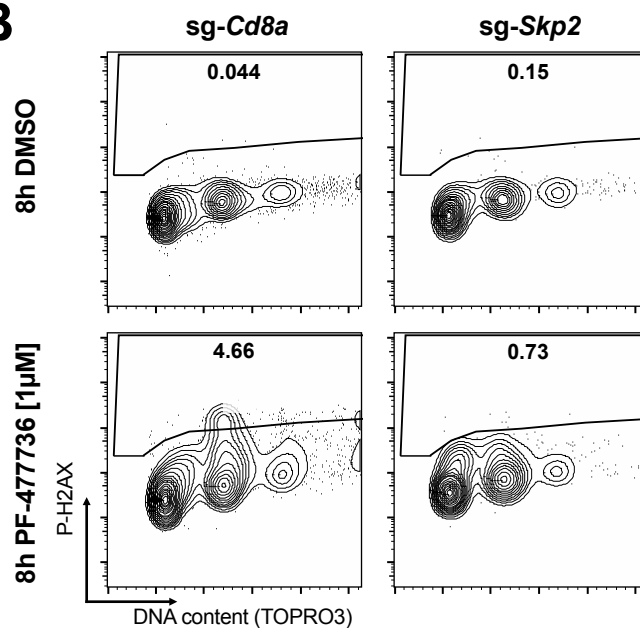**C**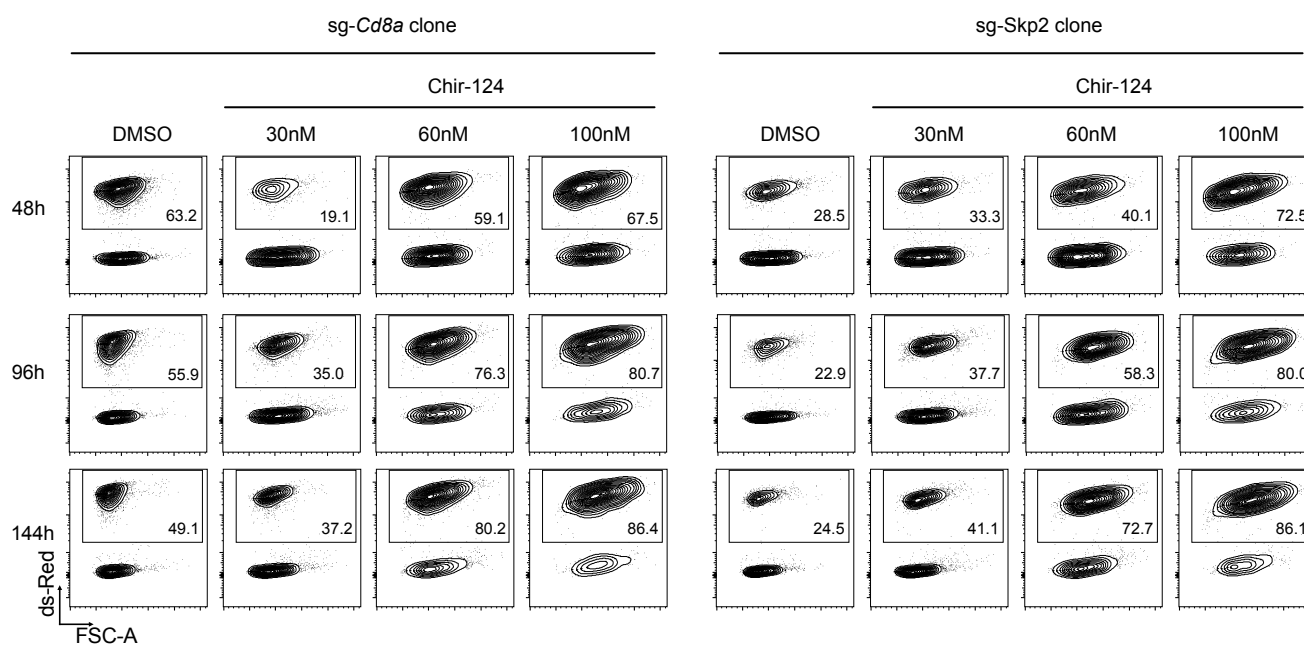**D**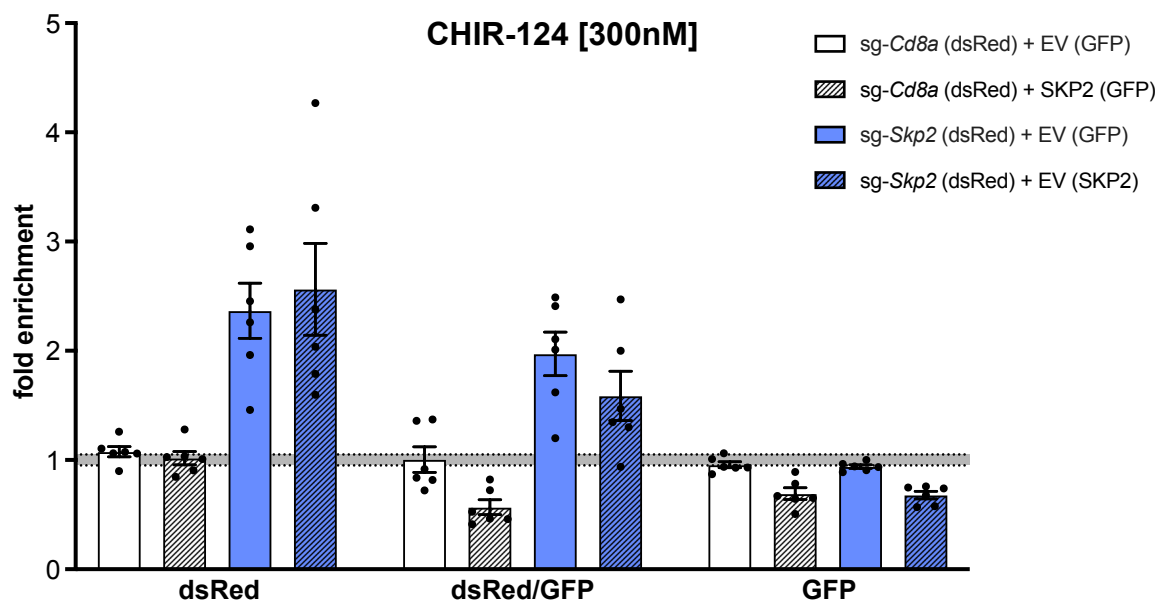

Supplement: Supplementary file 2 — Fig. S2. Exogenous SKP2 restores drug‐responsiveness. [file MOL2-16-2771-s001.pdf]

**A**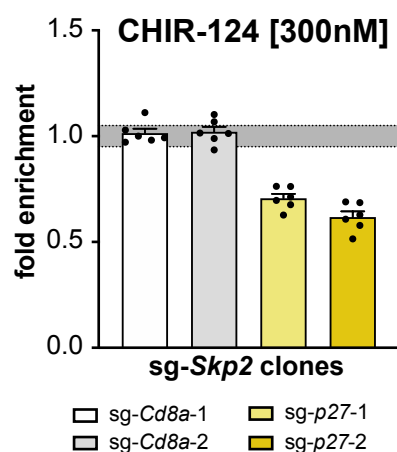**B**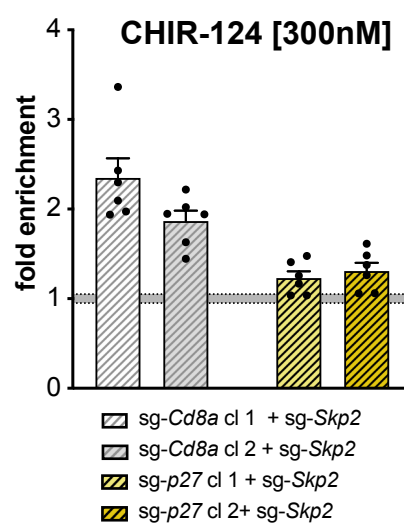**C**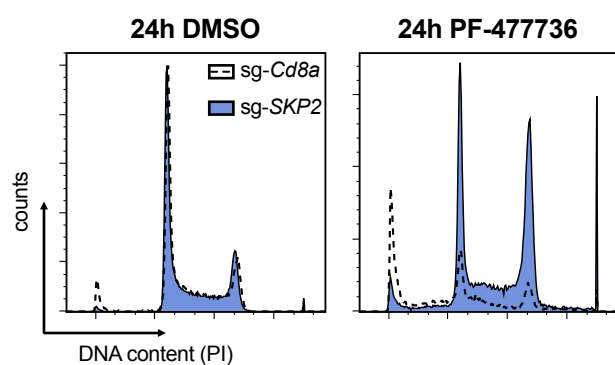**D**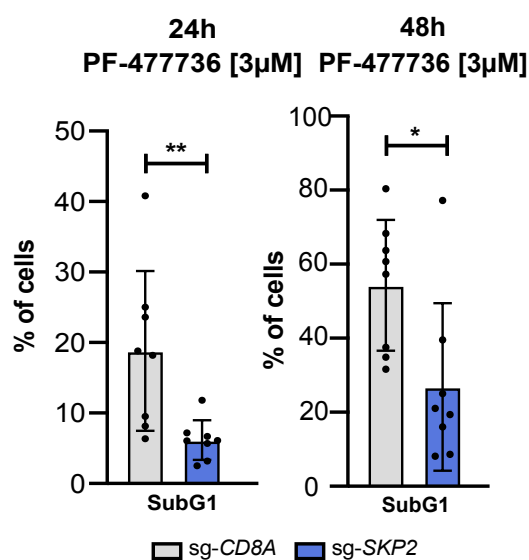**E**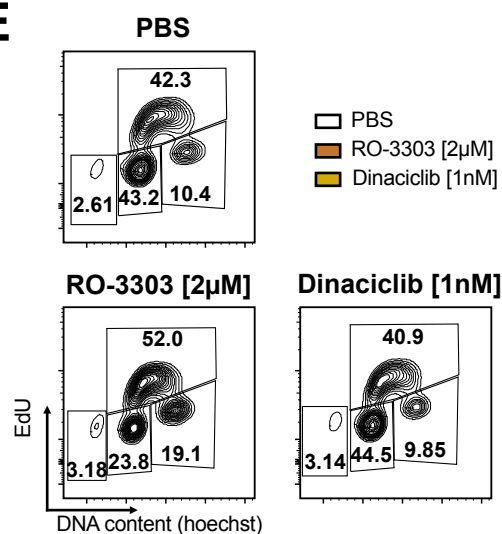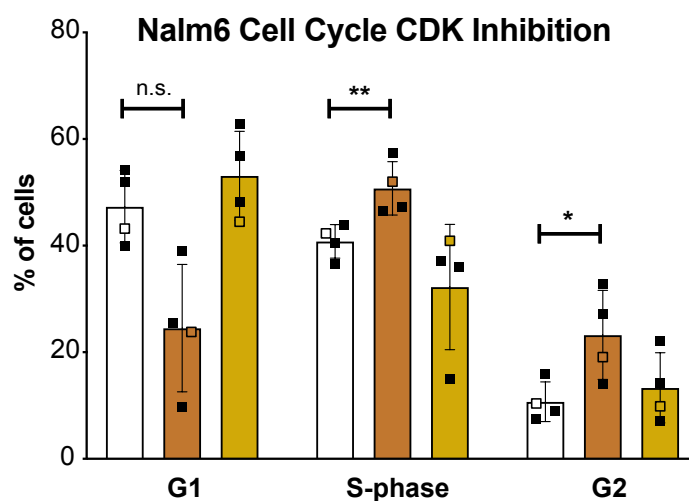

Supplement: Supplementary file 3 — Fig. S3. p27 is the key SKP2 substrate that defines CHK1i sensitivity. [file MOL2-16-2771-s003.pdf]
