# Supplementary material for: The SKP2‐p27 axis defines susceptibility to cell death upon CHK1 inhibition
Source: Mol Oncol. 2022 Jul 7;16(15):2771–87. doi: 10.1002/1878-0261.13264 (PMC9348596; doi:10.1002/1878-0261.13264)
Supplement: Supplementary file 4 [file MOL2-16-2771-s005.docx]

**Suppl. Figure legends:**

**Figure S1:** Identification of SKP2 as a modulator of CHK1i-induced apoptosis.

**(A**) Parental and BAX/BAK double knock-out (DKO) Baf3 cells were cultured for 48h in different concentrations of the CHK1-inhibitor CHIR-124. Cell death was assessed using PI staining and flow cytometry. Each bar represents the mean of the respective concentration (±SD) of six independent experiments (parental), five independent experiments (BAX/BAK DKO) (DMSO,250nM, 500nM, 1µM and 2µM) and two independent experiments for 125nM. **(B)** For validation, Baf3Cas9 cells were transduced with viruses encoding the sgRNA of interest and a dsRed marker gene (sgGOI/dsRed). sgRNAs targeting the *Cd8a* gene, not expressed in Baf3 cells, were used as controls. Three days post transduction cells were treated with the CHK1 inhibitor CHIR-124 [300 nM] or DMSO for 48h. The percentage of dsRed+ cells was assessed using flow cytometry, whereby the treatments were normalized to the DMSO-treated controls. Bars indicate the mean fold enrichment (±SEM) of three independent experiments. **(C)** Murine Hoxb8-FLT3 cells expressing Cas9 were transduced with viral particles encoding two different sg-*Skp2*/dsRed or sg-*Cd8a*/dsRed. Three days post transduction cells were treated for 48h with two different CHK1 inhibitors or DMSO as a control. The percentage of dsRed+ cells was assessed using flow cytometry, whereby the treatments were normalized to the DMSO-treated controls. Bars indicate the mean fold enrichment (±SEM) of three independent experiments. **(D)** Baf3 cells expressing pMIP-BCL2 (BCL2) or an empty vector (EV) control were transduced with two different sg-*Skp2*/dsRed constructs. Three days post transduction the respective bulks were treated with the CHK1 inhibitor CHIR-124 [300 nM] or DMSO for 48h. The percentage of dsRed+ cells was assessed using flow cytometry, whereby the treatments were normalized to the DMSO-treated controls. Bars indicate the mean fold enrichment (±SEM) of three independent experiments.

**Figure S2:** Exogenous SKP2 restores drug-responsiveness.

**(A)** Baf3 SKP2-KO and sg-*Cd8a* control clones (dsRed+) were transduced with human SKP2 (SKP2) or an empty vector (EV), which both express GFP as a marker. Three days post transduction cultures were treated with the CHK1i CHIR-124 [300 nM] for 48h. The percentage of GFP+ cells was assessed using flow cytometry. Treatments were normalized to the DMSO-treated controls. Bars indicate mean fold enrichment (±SEM) per genotype noted in three independent experiments (Cd8a-1, Skp2-1, Skp2-2) and two independent experiments (Cd8a-2). **(B)** Representative contour plots of Baf3 SKP2 KO and sg-*Cd8a* control clones treated for 8h with the CHK1 inhibitor PF-477736 [1 μM] or DMSO. DNA damage was assessed by γH2A.X^Ser139^and DAPI staining using flow cytometry. **(C)** Contour plots of Baf3 SKP2 KO and sg-*Cd8a* control clones (dsRed+) were mixed at a ratio of 15/85 with parental Baf3^Cas9^ cells and treated with low doses of the CHK1 inhibitor CHIR-124 for up to 7 days. The percentage of dsRed+ cells was assessed using flow cytometry. **(D)** Baf3 cells were transduced with two different sg-*Skp2*/dsRed or sg-*Cd8*a/dsRed constructs. Two days later the bulks were reconstituted with human SKP2 or an empty vector (EV), which both express GFP as a marker. Three days after the second transduction, cells were treated with CHIR-124 [300 nM] or DMSO for 48h. The percentage of all sub-populations was assessed using flow cytometry, whereby treated cells were normalized to DMSO controls. Bars indicate the mean fold enrichment (±SEM) of two individual sgRNAs targeting *Cd8a* or *Skp2* with three independent experiments.

**Figure S3:** p27 is the key SKP2 substrate that defines CHK1i sensitivity

**(A)** Baf3 SKP2-KO clones were transduced with two independent sg-*p27*/dsRed or sg-*Cd8a*/dsRed constructs, respectively. Three days post transduction the bulks were treated with CHIR-124 [300 nM] or DMSO for 48h. The percentage of dsRed+ cells was assessed using flow cytometry, whereby treated cells were normalized to DMSO controls. Bars indicate the mean fold enrichment (±SEM) of three independent experiments with two SKP2 KO clones. **(B)** Baf3 p27- and sg-*Cd8a* control clones were transduced with two independent sg-*Skp2*/dsRed constructs. Three days post transduction the cells were treated with CHK1 inhibitor CHIR-124 [300 nM] for 48h. The percentage of dsRed+ cells was assessed using flow cytometry, whereby treated cells were normalized to DMSO controls. Bars indicate the mean fold enrichment (±SEM) of three independent experiments (data of both clones werer pooled). **(C)** Representative DNA content histograms of a Nalm6 SKP2 KO and sg-*Cd8a* control cell line analyzed 24h after CHK1i or DMSO treatment. DNA content was assessed using PI staining and flow cytometry **(D)** Quantification of apoptotic subG1-cells after 24h or 48h of PF-477736 [3 µM] treatment. Bars represent mean percentage (±SD) of two repetitions in four clones per genotype. **(E)** Nalm6 cells were treated with RO-3303, Dinaciclib or DMSO for 8 hours. The cell cycle distribution of these cells was assessed via EdU incorporation for one hour and subsequent staining, followed by flow cytometry (EdU/DNA). Contour plots of cell cycle (left panel) and quantification of flow cytometry data (right panel). Data point of exemplary contour plot is highlighted in respective color. Bars represent average (±SD) of four repetitions. * p < 0.05; ** p < 0.01; *** p < 0.001; unpaired t-test.
